# Understanding AI-Assisted Care Technology Adoption Among Formal Care Providers: Insights From UTAUT

**DOI:** 10.1093/geroni/igaf122.1137

**Published:** 2025-12-31

**Authors:** Taekyung Kim, Donghun Kang, Jiwon Park, Moon Choi

**Affiliations:** Korea Advanced Institute of Science & Technology, Daejeon, Republic of Korea; Korea Advanced Institute of Science & Technology, Daejeon, Republic of Korea; Korea Advanced Institute of Science & Technology, Daejeon, Republic of Korea; Korea Advanced Institute of Science & Technology, Daejeon, Republic of Korea

## Abstract

This study examines the acceptance of four AI-assisted care technologies among formal care providers using the Unified Theory of Acceptance and Use of Technology (UTAUT). The Korean government has launched initiatives to develop and deploy care technologies to support independent living among older adults living alone. However, limited research has explored the intention of formal care providers—including care assistants, social workers, and visiting nurses—to adopt these technologies in delivering social and healthcare services to older adults. A total of 123 formal care providers, primarily care assistants (73.2% aged 50 or older; 92.7% women), participated in an online survey conducted in December 2024. The survey assessed performance expectancy, effort expectancy, social influence, facilitating conditions, and intention to use four technologies: (a) AI Care Call, (b) Emergency Alarm Service, (c) Companion Robots, and (d) Care-Net Platform. Path analysis revealed that technology acceptance varied across technologies. Social influence and effort expectancy were significant positive predictors of intention to use all four technologies, with social influence demonstrating the strongest effect. Performance expectancy was significantly associated only with the Emergency Alarm Service and Care-Net Platform. Given that formal care providers’ perceptions and intentions influence the successful deployment and adoption of care technologies among older adults, these findings highlight the critical roles of social influence—the perceived encouragement from significant others—and effort expectancy—the perceived ease of use—in facilitating adoption.

